# Reducing low-value imaging - stakeholders’ assessment of an intervention to improve imaging services

**DOI:** 10.1186/s12913-024-11648-y

**Published:** 2024-10-03

**Authors:** Elin Kjelle, Ingrid Øfsti Brandsæter, Eivind Richter Andersen, Bjørn Morten Hofmann

**Affiliations:** 1 Department of Health Sciences Gjøvik, Norwegian University of Science and Technology (NTNU), 2802 Gjøvik, Norway; 2 Centre of Medical Ethics at the University of Oslo, 0318 Oslo, Norway

**Keywords:** Low-value imaging, Radiology, Multicomponent intervention, Choosing wisely campaign, Evaluation

## Abstract

**Background:**

An intervention to reduce low-value magnetic resonance imaging (MRI) was designed and implemented in private imaging centres in Norway in October 2022. The intervention used return letters for poor referrals of MRI of the lower back, brain and knee at private imaging centres in Norway. The study aimed to investigate key stakeholders’ experiences and assessment of the intervention and the specific research questions were:

• How many return letters were sent during the study period?

• What were the medical directors’ and managers’ experiences with and reflection on success factors for the intervention implementation and using return letters?

**Methods:**

The number of return letters sent was collected directly from Norway’s two main private imaging providers. Two semi-structured individual interviews were conducted with the medical directors of the imaging providers, as well as two focus group interviews with nine managers from the various private imaging centres operated by the two imaging providers.

**Results:**

In total, 1,182 return letters were sent for patients undergoing one of the three types of MRI examinations, and the number of return letters was highest at the beginning of the intervention. The interview analysis resulted in five categories: general experience, anchoring, organisation, return letter procedure and outcome. Sufficient information, anchoring and support were identified as crucial success factors.

**Conclusions:**

This study provides insights into the practical and crucial details of implementing interventions to reduce low-value imaging. The intervention was generally well received, and the high initial number of return letters decreased rapidly over the course of the study. Several key success factors were identified.

**Supplementary Information:**

The online version contains supplementary material available at 10.1186/s12913-024-11648-y.

## Background

In modern healthcare, diagnostic imaging plays a key role in the diagnostics, prognosis and treatment of a wide range of diseases [1]. As diagnostic imaging is a shared and scarce resource, access to imaging can affect the quality of health services and patient care at all levels of healthcare provision. Therefore, diagnostic imaging should be used only when it is of high value to the patient [1].

Unnecessary use of diagnostic imaging has several potential negative consequences; Firstly, the patient is exposed to risks such as radiation [2] and might experience side effects from contrast media [3] and other medications related to the procedure. Secondly, false (positive and negative) test results, overdiagnosis and incidental findings can lead to delayed, unnecessary or even harmful treatment. Consequently, in the worst-case scenario, the patient may suffer from a treatment that was not necessary in the first place [4]. Thirdly, unnecessary diagnostic imaging utilisation represents a waste of healthcare resources [5].

One source of unnecessary imaging is low-value examinations, where “evidence suggests it confers no or very little benefit on patients, or risk of harm exceeds likely benefit, or, more broadly, the added costs of the intervention do not provide proportional added benefits” [6]. In imaging, several examinations have been identified as potentially low-value for specific patient groups or clinical problems [7–9]. Computed tomography (CT) of the head for minor head injuries, magnetic resonance imaging (MRI) of the lower back for back pain, MRI of the brain for headaches without red flags, and follow-up conventional radiography (CR) after knee surgery if the patient has a negative clinical examination [8, 9] are examples of imaging procedures that might prove unnecessary.

Consequently, several actions have been taken to reduce low-value imaging; for instance, developing and implementing guidelines for referrers, the use of clinical decision support, feedback to imaging providers and shared decision-making [10]. Moreover, multi-component measures seem to be most successful in reducing low-value imaging. However, there is still a significant overuse of diagnostic imaging worldwide [11].

The Choosing Wisely campaign is an ongoing initiative to reduce low-value health services [12]. Several national medical associations and other professional health organisations have joined the campaign [12] and identified imaging that is recommended not to be performed [9, 13–15]. The Norwegian Radiological Society has published six recommendations, three of which relate to MRI examinations often performed in private imaging centres in Norway, including imaging for lower back pain, imaging of the brain for headaches and advanced imaging in anterior knee pain for some patient groups [15], details are presented in Table 1.

**Table 1:** Details of the three recommendations from the Choosing Wisely campaign relevant to this paper

**Recommendations from the Choosing Wisely campaign**
Avoid diagnostic imaging for low back pain for adults without red flags. Examples of red flags: Fever or other signs of infection, history of injury or recent spinal puncture, and accompanying general symptoms.
Avoid advanced diagnostic imaging for anterior knee pain if the patient does not have hydrops or locking or has tried physical treatment without improvement. If uncomplicated disc herniation or uncomplicated spinal stenosis is suspected, imaging is only indicated after 4-6 weeks of conservative treatment and if surgery is being considered.
Avoid diagnostic imaging of the head for an uncomplicated headache unless any red flags are present. Examples of red flags: Rapidly increasing frequency and severity of headaches or lack of coordination.

In Norway, private imaging centres are partly commissioned by the health authorities to perform outpatient imaging on behalf of the authorities. In addition, they provide imaging for patients who pay out of pocket to be examined outside the public healthcare system. Two private imaging providers (companies) run the majority of private imaging centres in Norway. According to Norwegian law, patients must be referred to imaging by a physician/specialist, manual therapist (i.e. physiotherapists with expertise in musculoskeletal system) or chiropractor – and the radiologist can reject referrals where the examination is not justified [16]. This regulates both publicly and privately funded imaging services. Private imaging centres receive referrals from general practitioners, manual therapists, chiropractors, and private practising specialists.

Based on the Choosing Wisely campaign in Norway, an intervention to reduce low-value MRI imaging was designed and implemented in private imaging centres in 2022 [17]. The intervention targeted the three Choosing Wisely campaign recommendations mentioned above: MRI of the lower back, brain, and knee using referral return letters. The term referral return letter, or “return letter” in short, was used as this is close to the Norwegian meaning, emphasising that the return of the referral is not an absolute rejection of the imaging request. This means that when a referral is returned to the referrer, the patient does not get an appointment for imaging at this point. If the referrer sends more relevant information about the patient’s condition, the referral will be assessed again, and imaging could be scheduled if found justified. The intervention is described in more detail in a separate publication [17], and a summary of the intervention is presented in the Method section below.

This study is part of a more encompassing evaluation of various intervention outcomes through multiple methods and specifically focuses on the managers’ and medical directors’ experiences with and perspectives on implementing the intervention. As managers on all company levels hold an essential role in successfully implementing changes [18, 19], their experiences and reflections are vital as they help identify barriers and perspectives which are crucial when imaging departments want to implement interventions to reduce low-value imaging. An earlier publication has evaluated the staff and referrers’ perspectives of a pilot intervention in this project [20]. However, this study provides new knowledge as it explores managers’ perspectives on the full national implementation. Accordingly, this study aimed to explore the implementation of return letters to reduce low-value MRI of the lower back, brain and knee in private imaging centres in Norway. The specific research questions were:

- How many return letters were sent during the study period?
- What were the medical directors’ and managers’ experiences with and reflection on success factors for the intervention implementation and using return letters?

## Methods

### The implemented intervention

In 2022, an intervention to reduce low-value imaging was introduced in imaging centres operated by Norway’s two largest private imaging providers. The intervention included three steps and is explained in more detail in a paper by Hofmann et al. [17].

1. A procedure for referral assessment was developed for radiographers and radiologists to use when assessing referrals for MRI of the lower back, brain and knee. Radiographers and radiologists were informed of this procedure through scheduled meetings.
2. A ‘return letter’ was developed for each of the three MRI procedures to inform referrers of why the referral was returned, stating that they should submit a new referral if they had additional and relevant clinical information about the patient. Drafts of the return letters were sent for revision to radiologists and referrers in different parts of the country to ensure clarity in the final version (English version in Additional file 1).
3. A two-step information campaign was created a) to inform referrers of the implementation of the new procedure and the recommendations of the Choosing Wisely campaign via emails, newsletters and academic journals; and b) to inform patients about why MRI of the lower-back, knee, and brain often is unnecessary through informational videos in waiting rooms and articles in newspapers and online.

Two private imaging centres, one from each provider, started a pilot intervention in May 2022 [20]. During the pilot, there were 200 return letters for MRI of the lower back and 44 return letters for MRI of the head, while no letters were sent for MRI of the knee as the radiologists found the recommendation too diffuse and thus difficult to use [20]. After a short assessment period, the intervention was extended to all the providers’ centres in Norway (in total, 28 private imaging centres) from October 2022 until the end of June 2023. During the study period, approximately 5,000 monthly MRI examinations were performed on each of the three examinations: lower back, head and knee.

#### Return letters

The collected data included information about which return letter was used (i.e. MRI of lower back, head or knee), the imaging provider and centre, and the month/year the letter was sent (between 1 October 2022 and 31 June 2023). The total number of returned referrals during the study period was also collected. The IT departments at the two providers operating the private imaging centres supplied the data.

#### Interviews

Two individual semi-structured interviews and two semi-structured focus groups were conducted. The individual interviews included the medical directors (radiologists) in charge of the implementation at the respective companies. The research group contacted the two medical directors directly and asked them to participate in individual interviews, as they played a vital role in the implementation, different from the role of the other managers.

The focus group interviews included the managers (radiographers) holding the position of head of the local centres. They were responsible for implementing the intervention locally in each provider’s various private imaging centres. The two medical directors helped identify the managers from the local centres that had the most experience with and insights into the intervention. The research group sent invitation letters to the managers via email. In total, five participants from each provider agreed to participate. There was one focus group per provider to allow for discussions between the managers and to learn from potential differences between imaging centres within the same provider. All participants received an information letter and consent form via email. They returned the signed consent form before the interview commenced.

Two slightly different interview guides (Table 2) were developed and piloted – one for the individual interviews and one for the focus groups. The semi-structured approach was chosen to ensure that the same topics were discussed with all participants while also allowing for relevant topics to be openly discussed [21]. The topics discussed in the interviews were experiences with implementing return letters, planning, information, training, and the reactions of employees and referrers.

**Table 2:** Outline of interview guides – main topics and relevant follow-up topics

**Individual interview**	**Focus group interview**
*What is your experience of the process of implementing a return letter in the Choosing Wisely campaign recommendations?*	*We would like you to discuss your experiences implementing return letters in the Choosing Wisely campaign recommendations.*
◦ Anchorage in the organisation and top management	◦ Information from top management
◦ Experiences from the pilot	◦ Training
◦ Feedback from employees	◦ Employees’ feedback/reactions
◦ Planning	◦ Reactions among referrers
◦ Cost/effectiveness	◦ Usefulness
◦ Evaluation	◦ Challenges
◦ Challenges	◦ Local adjustments
*Do you plan to continue using return letters, and if so, how and why?*	*Do you plan to continue using return letters, and if so, how and why?*
◦ Routines/procedures	◦ What needs to be in place?
◦ Training/follow-up	

The interviews were conducted between February and June 2023. Since the respondents live in different parts of the country, the interviews were conducted via video conferences (Zoom Video Communications, Inc., San Jose, USA) to avoid extensive travel. A digital audio recorder was used to record the dialogue from the interviews, which were then transcribed verbatim and anonymised by giving each participant a number.

All authors were involved in the intervention’s planning and evaluation. However, they were not hands-on in implementing the intervention in clinical practice. EK (radiographer and experienced researcher) conducted the focus group interviews, with BMH (professor and experienced researcher) as an observer and notetaker. EK and BMH each conducted one individual interview with the medical directors. EK and BMH transcribed the interviews they led.

### Analyses

Quantitative data on return letters was analysed with basic descriptive statistics in Excel.

**Fig. 1:**
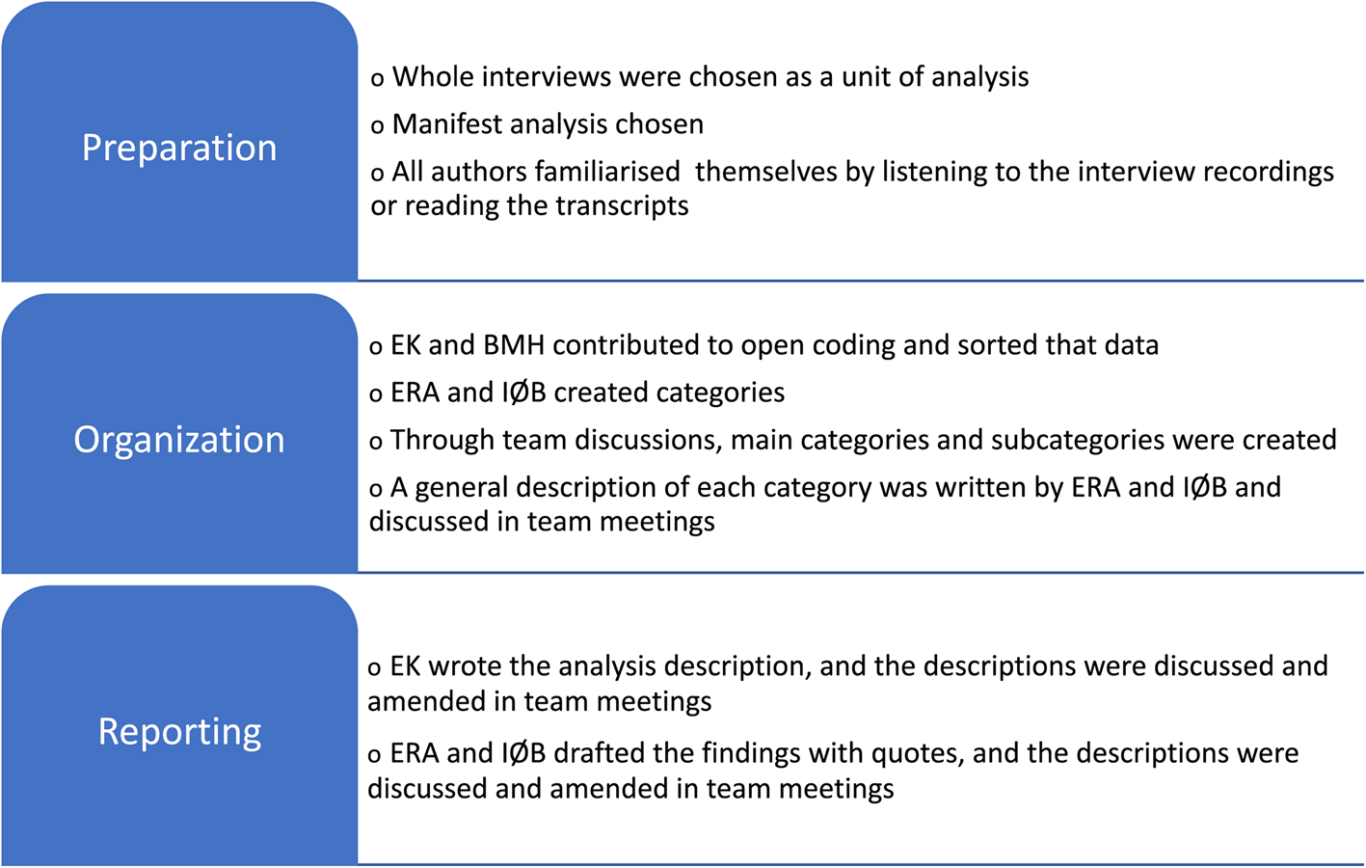
An illustration of the actions performed in the three phases of content analysis according to Elo and Kyngäs [22]

Data from the focus group and individual interviews were analysed together using inductive content analysis based on Elo and Kyngäs [18]. The analysis was conducted in three phases: preparation, organisation, and reporting. Figure 1 illustrates the analysis, and Table 3 gives an example of the analysis.

**Table 3:** An example from the analysis

**Summary**	**Condensed text**	**Key element**	**Category**
It is anchored in the management, which is necessary. We had several meetings during the planning process and discussed this in the mid-level manager meetings. I think it was important that the local managers be able to give their input.	Anchoring in management Discussions with mid-level management and local manager’s opinion are crucial.	Anchoring in management is crucial.	Anchoring

### Ethics

The Norwegian Agency for Shared Services in Education and Research approved the processing and storage of personal information in this study (Ref. 974,188).

## Results

### Return letters

**Fig. 2:**
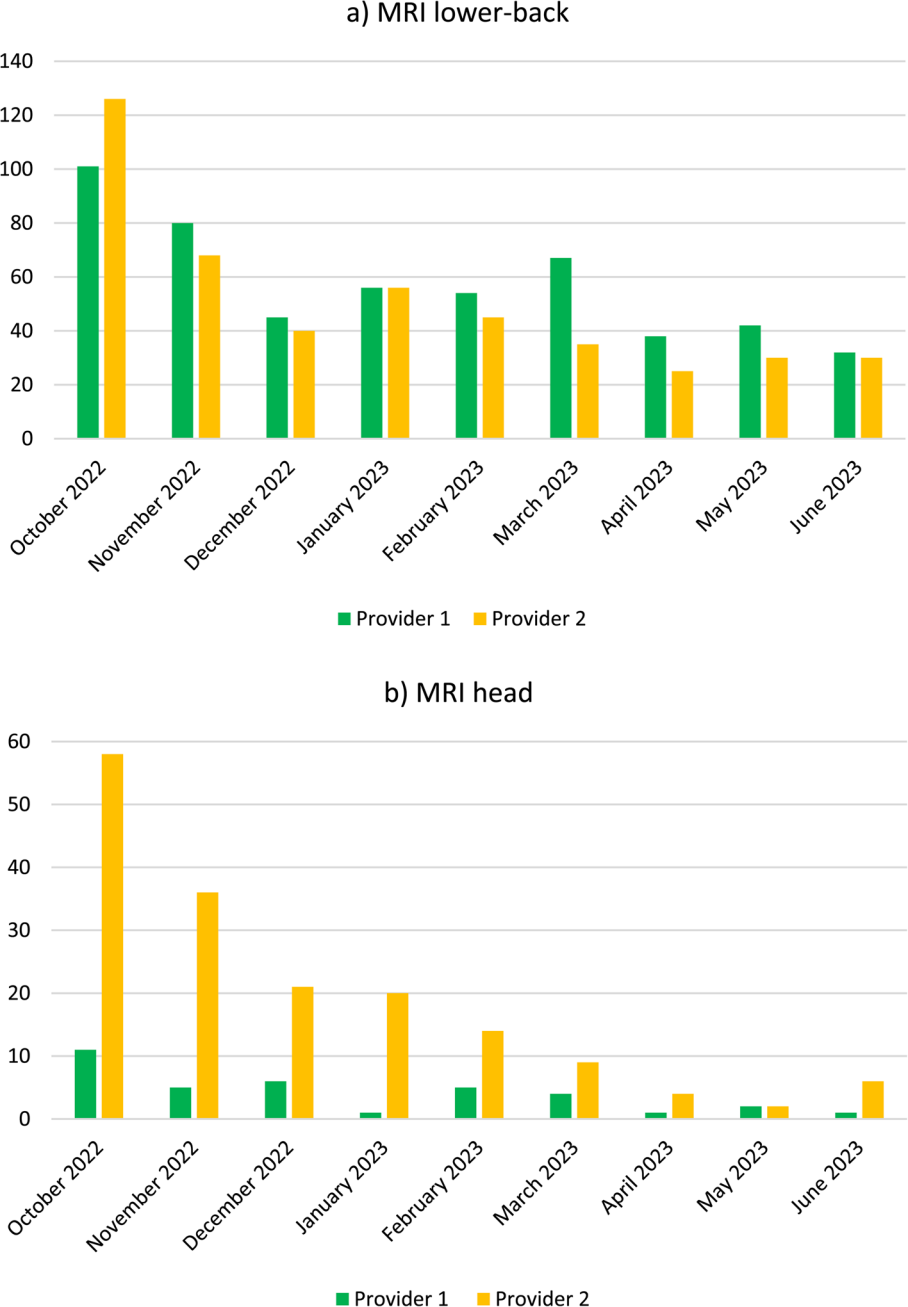
**a** and **b** The number of return letters sent for MRI of the lower back (**a**) and MRI of the brain (**b**) from all private imaging centres of the two imaging providers during the study period from May 2022 to June 2023. The y-axis is the number of return letters sent. MRI: Magnetic resonance imaging

In total, 4,375 referrals for the three types of MRI examinations were returned from all centres included in the study between October 2022 and June 2023. Of these, 1,260 were return letters related to this project, 970 were for MRI of the lower back, and 290 were for MRI of the head. There were some differences between the two imaging providers. In addition, there was a wide variation in return letters sent between the private imaging centres within the imaging providers, ranging from zero to 46 return letters sent in one month. Figure 2 shows the variation in return letters during the study period and the variation between the two imaging providers. Only six return letters were sent for knee MRIs from October 2022 through June 2023, these are not included in the figure as there are so few.

### Managers’ experiences

The four interviews lasted 45 min on average (27–57 min). The analysis yielded five categories: general experience, anchoring, organisation, return letter procedure, and outcome.

#### General experience

The general experience emphasised by both the medical directors and managers was that their staff and referrers received the intervention well. The managers stated that returning unwarranted referrals was common practice before launching the intervention, and that they therefore expected to receive negative comments when referrals were returned. However, the managers reported to receive fewer negative comments from referrers who received return letters than expected. One medical director suspected that some negative referrers did not read the return letter thoroughly before complaining. One manager said:

Some referrers’ feedback suggested they had not read the return letter properly. They had just gotten a little worked up and sent a new referral or inquiry.

Even though returning referrals were a standard practice at most private imaging centres, the practice of a standard referral assessment procedure was new. However, the participants stated that referrals often lack essential information, leading to poor referral quality, described as a barrier to a good referral assessment. This was perceived as a well-known problem, and the participants agreed that measures should be implemented to address this problem. One medical director stated:

I have been frustrated with poor-quality referrals for a long time (…). In my opinion, to get better referrals, there need to be consequences [for the referrer when the quality is bad].

#### Anchoring

The medical directors deemed support and anchoring within the organisation to create a shared understanding of the intervention and the need for the changes to be an important intervention success factor both organisationally across the healthcare system and locally at the private imaging centres.

At the organisational level, it was considered essential that both imaging providers followed the same procedure and that the intervention was implemented simultaneously by both imaging providers, yielding the same return practice across the country. Thus, when receiving a return letter, a referrer could not undermine the intervention by referring to the competing imaging provider.

Further, the medical directors emphasised the importance of solid anchoring to top-level management. One medical director said:

It [the intervention] is anchored with top management, which is essential (…) as we must make choices where we could lose money but where the quality of the service is more important.

At the local level, the procedure and return letter had to be integrated into the IT system to make the interventions work. Accordingly, regarding adequate IT systems and protocols, the participants deemed the intervention well-prepared before the start. One of the medical directors stated that the evaluation of the pilot intervention demonstrated the anchoring of the intervention across the organisation, and this made them more confident in launching the intervention on a national level. One medical director said:

They [the staff] found it meaningful to participate in the intervention (….). Then, I was even less worried about implementing the intervention in the rest of the country.

No public hospitals participated in the intervention, and one of the medical directors experienced a lack of support from the public hospitals. It was experienced as frustrating that not all imaging providers followed the same procedure. On the other hand, some local managers received positive feedback from public hospitals regarding the private centres’ involvement in the intervention. One manager stated:

We cooperate closely with the local hospital, and they appreciate us taking part in this intervention.

#### Organisation

The participants also emphasised the importance of organisation and described how the intervention was organised across the two imaging providers and locally within the private imaging centres. The medical directors emphasised the importance of keeping the financials separate from the clinical work. The referral assessment staff had no economic incentives or responsibilities related to the centres’ income. One medical director stated:

The radiologists and radiographers [assessing referrals] have nothing to do with the reimbursement or the money (…), which could affect the choice [when assessing referrals], but it does not as we make sure these roles are separated.

One aspect considered crucial to successfully implementing the intervention within the organisation was ensuring adequate information distribution. The medical directors highlighted the importance of informing all staff members about the intervention – both those directly affected by the intervention and those only indirectly involved. Information was given in department meetings. However, the medical directors experienced that it was difficult to be hands-on and to give enough information to everyone within an organisation with multiple locations across the country. Therefore, the responsibility to train, inform and encourage staff members to carry on with the intervention was given to the local managers. One medical director said:

As we have private imaging centres nationwide, I found it difficult to be hands-on. I have tried, but some things drown in everyday tasks, and then things run their own course.

The managers had varying experiences regarding information about the intervention. Some said they received adequate information before the intervention started, while others wanted more information about the background of the intervention. The managers’ statements highlighted that the responsibility of referral assessment and sending return letters varied across locations. At some centres, the radiographers assessed referrals without involving the radiologists, only consulting a radiologist when a referral was deemed challenging to assess. At other centres, the radiographers felt insecure and reported that they needed more information about the intervention to assess the referrals. Therefore, they left the evaluation of referrals to the radiologists. One manager said:

I suppose it’s about how we dealt with the insecurity among the radiographers internally. In retrospect, I could have used more time to make them [the radiographers] feel secure, but we chose to let the doctors handle it [the referral assessment].

Technical solutions and their use varied across locations, influencing the organisation of who could return referrals. Some managers described challenges related to the technical solutions enabling radiographers to send return letters, which led to various solutions. At some centres, the radiographers were permitted to send answers directly to the referrers, while others required a workaround. At one centre, the radiographers sent their assessments to a secretary, who then sent the answers to the referrers. In contrast, other radiographers sent their assessments to the radiologist, who sent the return letters to the referrers with a single keyboard stroke. The radiologists assessed all referrals at other centres because it was deemed the easiest solution. One manager said:

We radiographers want to do it [assess referrals], but we haven’t figured out the technical solutions yet.

#### Return letter procedure

The managers and medical directors agreed they were more thorough and returned more unwarranted referrals at the beginning of the intervention. One manager stated:

Our experience is that we were much better at returning referrals at the beginning of the intervention. We probably have a potential for improvement there.

Even though most managers were satisfied with the return letters, not all criteria for the three MRI examinations were equally easy to apply. It was deemed easier to assess and return referrals for MRI of the lower back than the brain or knee. The managers described the criteria for lower back pain as ‘good and easy to understand’, while the criteria for MRI of the brain and knee were not perceived as clear. In addition to poor referral quality, the managers described the use of the criteria as ‘challenging ‘ because it could be vague. One medical director said:

I think the criteria for MRI of the lower back works relatively well. For knee MRIs, it doesn’t work at all (…), and the problem with MRI of the brain is that it can be something very severe, right, that can’t wait.

Still, the medical directors and managers said that the return letters used in the intervention were well-written, highlighting that it was helpful to have standardised letters to send when returning a referral. However, some managers stated that the radiologists wanted the opportunity to individualise their responses to the referrer. They stated that some radiologists may have deviated from the return letters and provided their own written responses instead. Therefore, these returns will not have been registered within the procedure of the intervention and may yield an underestimation of the returns during the study period. One manager said:

One comment from the radiologists in my team is that they wanted to adjust the answer that was sent with the returned referral. So many of the returns may not be registered as part of the intervention.

Also, the referral process was influenced by communication between the referrers and private imaging centres. The managers described an overall good relationship with the referrers. However, the managers’ previous experiences with temperamental referrers might influence the referral assessment process. Some managers said that they would more readily accept the referral if the referrer were known to complain. One manager said:

When you remember the name [of an angry referrer] and receive a new referral, you tend to judge the referral towards acceptance.

#### Outcome

The managers described one outcome of the intervention as re-sending returned referrals with more information about the patient, making it easier to assess the referral accurately. Furthermore, one manager stated that the radiologists found using the standardised return letter helpful. Accordingly, the managers and the medical directors agreed that they wanted to continue using standardised return letters after the intervention ended. The medical directors also said they wanted to standardise return letters for other examinations to ensure that referrers understand why the referrals were returned – and learn from it. The managers discussed the intervention as an important step in taking pride in their work tasks. One manager said:

I think we should ‘choose wisely’ on everything [referrals] we receive. We should generally take professional pride in all we receive and deliver. We have to take that with us further, no matter what it is called: ‘Choosing Wisely’ or ‘justification’.

The managers stated that the fact that the private imaging centres were a part of this intervention had improved their reputation, and they believe that this would help the private imaging centres to be taken more seriously in the future. In addition, the managers experienced it beneficial that the intervention was developed and evaluated in cooperation with a neutral, external research team, increasing the perception that quality of service and medical practice, rather than financial gain, was in focus. The managers talked about how referrers now can see that the private imaging centres want to do a good job, are serious about their responsibility in justification assessment and are not solely focused on making money by performing as many examinations as possible. One manager said:

It has generated some positive articles in the media, where new, experienced and retired referrers comment that it is nice to see private imaging providers work this way. It has contributed to a better reputation for us [private imaging providers].

## Discussion

This study investigates the medical directors’ and managers’ experiences with an intervention to reduce low-value MRI imaging by private imaging providers in Norway regarding the reception of the intervention and the success factors for its implementation. The findings show that the initial high number of return letters rapidly decreased throughout the study. The general experience amongst managers and medical directors from both imaging providers was that the intervention was well received by both referrers and the centres’ staff. However, there were some marked exceptions, for instance, the participants reported that the private imaging centres organised the intervention differently.

The return letter approach is a multicomponent intervention, which is the type of intervention with the highest success rate in reducing low-value imaging [10]. Assessing the targeted examinations, involving stakeholders, and identifying and adjusting facilitators and barriers [23–29] were key factors in designing a context-sensitive intervention. However, Fig. 2a and b show that the number of return letters was highest in the first month of the intervention and lowest during the last months. This aligns with the participant statements that return letters were most used at the beginning of the intervention. Moreover, we found substantial differences in the number of return letters sent from the various private imaging centres operated by the two imaging providers. The referral assessment process was described as being adapted to the local context. However, differences in organisation can be one explanation for the variation between centres. Another reason can be outer context characteristics such as socioeconomic status and patient volume [30].

There may be many reasons for the reduced number of return letters over time:

1. Improved quality of the referrals as the return letters had a learning effect on the referrers. The respondents pointed out that poor referral quality was the main driver of return letters and potential subsequent frustration. The participants experienced referrers re-sending returned referrals with more information included, resulting in an easier assessment. However, Kanaan et al. [31] implemented an educational intervention for appropriate utilisation of CT pulmonary angiography and found no change in the appropriateness after the intervention. They suspect that a repetition of the educational intervention may have changed the outcome as it had in another study [32].
2. ‘Wear off’, as the intervention might lose attention and implementation fatigue may appear, resulting in lower compliance with the guidelines.
3. Other individually tailored returns of referrals, which were not part of the intervention, were used instead of standard return letters; and
4. The referrers adjusted to the referral requirements by adding false information to get the right criteria (i.e. ‘referral creep’).

The intervention’s effect on referral quality is the topic of a separate (ongoing) study and is beyond the scope of this article. The present study revealed that the participants experienced receiving adequate information before the intervention started, while some wanted more information about the background of the intervention. This is not in line with the findings of the pilot evaluation, where the participants said they received enough information before it began [20]. The difference between the pilot and the national intervention can be due to proximity and the intensity of information (described below). Training, continued access to information, and knowledge are important factors to manage when adopting an intervention to reduce low-value care [19, 30].

The participants described the criteria for MRI of the knee and brain as unclear and challenging to use. Lack of information and support for how to use the criteria for these types of MRI can explain the differences in number of return letters sent for the three MRI examinations [19]. E.g. the return letter for MRI of the knee were not used, while several return letters for MRI lower-back were sent. The criteria for lower back pain were characterised as being clearest. Moreover, the private imaging centres returned several referrals for MRI of the knee for reasons other than those given in the standard return letter.

Additionally, the medical directors stated that they found it difficult to be hands-on and give enough information to everyone because the private imaging centres were distributed across the country. The medical directors could not be present at all private imaging centres due to the vast distances between them. Interventions and innovations are more likely to become routine if the management is actively involved [19]. However, the middle managers are often not included in research about implementations of intervention, and Birken et al. [18] suggest that including the managers in implementations may increase the effectiveness of healthcare interventions. In addition, managers must communicate with the top management about what type of support they need to improve their commitment to the intervention [33]. Thus, differences in management involvement can be one reason for the differences between the pilot and the full rollout of the intervention.

The organisation’s values and goals are also factors that influence the adoption of an intervention [19]. The medical directors said that the intervention heightens the reputation of private imaging providers and, hence, their value and the value of their work. Thus, they stated that they wanted to continue using the return letters after the intervention had ended.

### Strengths and limitations

This study only includes managers and medical directors in the interviews. Interviewing staff and the referrers would add to the evaluation. However, this study aimed to explore the responses and experiences in the centres where the intervention was implemented. The referrers’ experiences merit a separate study.

One strength of this study was the use of focus group interviews. The focus groups with managers from different private imaging centres made it possible for the managers to be aware of and discuss how the centres chose to organise the intervention differently and share experiences.

While this study has focused on the responses to and experiences with implementing return letters for specific low-value examinations, subsequent studies will focus on other outcome measures, such as the quality of referrals. This study’s value is that it provides insights into the practical and crucial details of implementing interventions to reduce low-value imaging.

Further research is needed to evaluate the effect of the intervention on the number of low-value imaging sessions conducted compared to how many referrals were returned. In addition, exploring the impact on referral quality with more relevant patient information would be interesting, hence investigating if the intervention had a learning effect on the referrers.

## Conclusions

This study provides insights into the practical and crucial details of implementing interventions to reduce low-value imaging. The number of return letters sent was highest at the beginning of the study, with a rapid decrease over the entire period. The overall experience amongst managers and medical directors was that the intervention was well received by the centres’ staff and referrers, with some exceptions. Sufficient information, anchoring and support were identified as crucial success factors for the intervention to reduce low-value imaging.

## Supplementary Information

Additional file 1.

## Data Availability

The datasets generated and analysed during the current study are not publicly available due to individual privacy conditions but are available from the corresponding author upon reasonable request.

## Abbreviations

CR: Conventional radiography
CT: Computed tomography
CTPA: CT pulmonary angiography
MRI: Magnetic resonance imaging
