# Supplementary material for: Reducing low-value imaging - stakeholders’ assessment of an intervention to improve imaging services
Source: BMC Health Serv Res. 2024 Oct 3;24:1170. doi: 10.1186/s12913-024-11648-y (PMC11448175; doi:10.1186/s12913-024-11648-y)
Supplement: Supplementary file 1 — Additional file 1. [file 12913_2024_11648_MOESM1_ESM.pdf]

Additional file 1. Return letter

Return of referral to MRI for lower-back pain or headache.

On 1 May, imaging centres employed a new procedure for referral assessment related to specific examinations included in the "Choosing Wisely campaign," meaning that referrals not in line with the recommendation developed by the Norwegian Society of Radiologists will be returned. The purpose of the new procedure is to remind practitioners of good clinical practice and raise their awareness of this. In the long term, this will increase resource utilization and reduce waiting times.

The indication for MRI-examinations for lower-back pain or headache is assessed by the following criteria:

Examination	Problem	Indicated if	Red flags
Lower-back MRI	Lower-back pain	○ Conservative treatment and considering surgery.<br><br>○ Duration < 4-6 weeks **with** red flags<br>OR<br>○ Duration> 4-6 weeks	○ Fever<br>○ Sign of infection<br>○ Trauma<br>○ Recent spinal puncture<br>○ Accompanying impaired general condition<br>○ Increased urination difficulties<br>○ Neurologic deficit
Brain MRI	Headache	○ Headache with red flags	○ Sudden debut<br>○ Rapidly increasing frequency and severity<br>○ Headache that wakes the patient from sleeping<br>○ Dizziness<br>○ Loss of coordination<br>○ New neurological symptoms<br>○ Previous cancer or immunodeficiency<br>○ Paresthesia<br>• Burning<br>• "Pins and needles"<br>• Prickling<br>• Itching<br>• Numbness<br>○ Skin crawling

Based upon the referral we received, the problem described is perceived as not indicated by the professional environment. We are therefore returning the referral in accordance with the Radiation Protection Regulation section 39, Justification.

We apologise for the inconvenience.

If there is clinical information of importance not described in the referral, or if symptoms/duration imply that the examination should still be performed, we kindly request you to re-refer the patient with the necessary information in the referral.

Best Regards

References:

Choosing Wisely – Radiology

https://beta.legeforeningen.no/foreningsledd/fagmed/norsk-radiologisk-forening/artikler/fag-ogutdanningsstoff-fra-noraforum/gjor-kloke-valg-radiologi/

Radiation Protection Regulation:

https://lovdata.no/forskrift/2016-12-16-1659
